# Annexin V^+^ Microvesicles in Children and Adolescents with Type 1 Diabetes: A Prospective Cohort Study

**DOI:** 10.1155/2020/7216863

**Published:** 2020-03-30

**Authors:** Vibeke Bratseth, Hanna D. Margeirsdottir, Gemma Chiva-Blanch, Martin Heier, Svein Solheim, Harald Arnesen, Knut Dahl-Jørgensen, Ingebjørg Seljeflot

**Affiliations:** ^1^Center for Clinical Heart Research, Department of Cardiology, Oslo University Hospital Ullevaal, Oslo, Norway; ^2^Faculty of Medicine, University of Oslo, Oslo, Norway; ^3^Pediatric Department, Oslo University Hospital Ullevaal, Oslo, Norway; ^4^Oslo Diabetes Research Centre, Oslo, Norway; ^5^Cardiovascular Program ICCC, Institut de Recerca Hospital Santa Creu i Sant Pau-IIB Sant Pau, Sant Antoni Maria Claret, 167, 08025 Barcelona, Spain; ^6^Endocrinology and Nutrition Department Institut d' Investigacions Biomediques August Pi Sunyer (IDIBAPS), Hospital Clinic, Barcelona, Spain; ^7^Centro de Investigacion Biomedica en Red Fisiopatologia de la Obesidad y Nutrition (CIBEROBN), Instituto de Salud Carlos III (ISCIII), Spain

## Abstract

**Background:**

Type 1 diabetes is a chronic disease including hyperglycemia and accelerated atherosclerosis, with high risk of micro- and macrovascular complications. Circulating microvesicles (cMVs) are procoagulant cell fragments shed during activation/apoptosis and discussed to be markers of vascular dysfunction and hypercoagulability. Limited knowledge exists on hypercoagulability in young diabetics. We aimed to investigate cMVs over a five-year period in children/adolescents with type 1 diabetes compared with controls and any associations with glycemic control and cardiovascular risk factors. We hypothesized increased shedding of cMVs in type 1 diabetes in response to vascular activation.

**Methods:**

The cohort included type 1 diabetics (*n* = 40) and healthy controls (*n* = 40), mean age 14 years (range 11) at inclusion, randomly selected from the Norwegian Atherosclerosis and Childhood Diabetes (ACD) study. Citrated plasma was prepared and stored at -80°C until cMV analysis by flow cytometry.

**Results:**

Comparable levels of Annexin V (AV^+^) cMVs were observed at inclusion. At five-year follow-up, total AV^+^ cMVs were significantly lower in subjects with type 1 diabetes compared with controls; however, no significant differences were observed after adjusting for covariates. In the type 1 diabetes group, the total AV^+^, tissue factor-expressing AV^+^/CD142^+^, neutrophil-derived AV^+^/CD15^+^ and AV^+^/CD45^+^/CD15^+^, and endothelial-derived AV^+^/CD309^+^ and CD309^+^/CD34^+^ cMVs were inversely correlated with HbA1c (*r* = ‐0.437, *r* = ‐0.515, *r* = ‐0.575, *r* = ‐0.529, r = ‐0.416, and *r* = ‐0.445, respectively; all *p* ≤ 0.01), however, only at inclusion. No significant correlations with cardiovascular risk factors were observed.

**Conclusions:**

Children/adolescents with type 1 diabetes show similar levels of AV^+^ cMVs as healthy controls and limited associations with glucose control. This indicates that our young diabetics on intensive insulin treatment have preserved vascular homeostasis and absence of procoagulant cMVs.

## 1. Introduction

Type 1 diabetes is a chronic disease leading to hyperglycemia and high risk of developing microvascular and macrovascular complications [1]. The altered glucose metabolism in type 1 diabetes changes the vascular homeostasis by increased production of reactive oxygen species and reduced nitric oxide (NO), favoring endothelial dysfunction (ED) and a proinflammatory milieu. Activated endothelial cells further attract monocytes and become more permeable, which accelerates the process of atherosclerosis. In diabetes, hyperglycemia contributes to procoagulant activity and increased risk of atherothrombosis via platelet hyperreactivity, enhanced transcription and activation of coagulation factors, and glycation of fibrinolytic proteins [2–6]. In type 1 diabetes subjects, cardiovascular events like myocardial infarction, stroke, and peripheral artery disease appear approximately 10-15 years earlier than in healthy controls and represent the main reasons for morbidity and mortality in these patients [7].

Circulating microvesicles (cMVs) are extracellular and heterogenous particles shed from the plasma membrane surface on cell types like platelets, leukocytes, monocytes, neutrophils, and endothelial cells [8]. They are released upon specific (cytokine activation, apoptotic cell blebbing, and coagulation) and nonspecific (high shear stress) stimuli, which activate calcium-dependent signaling and rearrangements of the cytoskeleton [9]. The cMVs express surface receptors and antigens according to their parental cell, and they are filled with lipids, genetic material, and different proteins [10]. These submicron particles participate in vascular homeostasis and intercellular communication during physiological conditions [11].

In disease states, the release of cMVs increases and the proportion that is expressing phosphatidylserine (PS) and/or tissue factor (TF) is highly procoagulant [12, 13]. Flow cytometry is the gold standard detection method, and procoagulant cMVs can be visualized by adding Annexin V (AV^+^), a high-affinity ligand for PS. cMVs can mediate disease progression by vascular injury, inflammation, oxidative stress, and hypercoagulability, and they have emerged as cardiovascular risk factors [14, 15]. cMVs from endothelial origin have been shown to associate with endothelial dysfunction [16, 17], arterial stiffness [18], and increased risk of cardiovascular disease (CVD) [19]. In patients with type 1 diabetes, elevated levels of total and endothelial- and platelet-derived cMVs have been demonstrated [20].

Cardiovascular risk factors are more commonly seen in children and young individuals with type 1 diabetes compared with the general pediatric population [21]. However, the complete understanding of the increased risk of CVD in type 1 diabetes is yet not fulfilled.

The aims of the current study were to investigate the presence of AV^+^ cMVs, from different vascular origins, in children and adolescents with type 1 diabetes compared with healthy control subjects over a five-year period, and to assess associations with glycemic control and cardiovascular risk factors in the type 1 diabetes group. We hypothesize higher levels of AV^+^ cMVs in the type 1 diabetes group than the healthy controls at both time points and a more pronounced increase in the type 1 diabetes group after five years, due to accelerated cell activation and disease progression. Furthermore, that the amount of AV^+^ cMVs is associated with glycemic control and traditional cardiovascular risk factors.

## 2. Methods

### 2.1. Study Population

The present cohort (*n* = 80: type 1 diabetes (*n* = 40) and controls (*n* = 40)) is a subgroup of the Norwegian Atherosclerosis and Childhood Diabetes (ACD) study, a prospective study on development of atherosclerosis in childhood onset type 1 diabetes with follow-up every fifth year [5]. At study baseline in 2006, all children and adolescents aged 8-18 years with type 1 diabetes, registered in the national Norwegian Childhood Diabetes Registry (NCDR) from the South-East health region in Norway, were invited to participate. The exclusion criteria were another serious chronic disease or pregnancy. The initial cohort consists of 314 type 1 diabetes patients (response rate of 40%), as well as 120 healthy control subjects that were friends of the diabetic patients, of similar age, and from the same milieu. All participants were encouraged to take part in the scheduled follow-up. According to the NCDR and conversation with the participants and parents, approximately all children and adolescents with type 1 diabetes (97%) were on intensive insulin treatment from the time of diagnosis, using either insulin pumps or basal-bolus regimens with > four daily insulin injections. Micro- or macrovascular complications were not present at either time point, also confirmed by the urinary albumin-creatinine ratio (U-ACR). All participants, as well as parents of those below 18 years of age, signed written informed consent to participate. Both the enrollment (2006-2008) and the five-year follow-up (2011-2013) were performed at the pediatric department, Oslo University Hospital, Ullevaal. The data collection was performed by the study nurse at both time points with standardized methods when possible. Anthropometric data, time of disease onset, and medication were registered in a case report form. The subgroup for the present investigation was randomly selected from the cohort. The National Committee for Research Ethics in Norway and the Norwegian Social Science Data Services endorsed the study protocols, and the project was performed according to the Declaration of Helsinki.

### 2.2. Laboratory Measurements

Fasting blood samples were drawn between 07.30 and 10.00 am at inclusion and after five years, in both study groups. Citrated plasma (3.8% sodium citrate) were removed from the blood cells within 30 min by centrifugation 2500 × g for 20 min at 4° C and then immediately frozen and stored at -80°C until further preparation for cMV analysis. HbA1c was measured at a Diabetes Control and Complications Trial-standardized laboratory by high-pressure liquid chromatography (Bio-Rad, Richmond, CA, USA), with a coefficient of variation < 3%. U-ACR was calculated from spot urine. Routine laboratory analyses were analyzed by conventional methods, and arterial blood pressure was measured according to the National High Blood Pressure Educational Program Working Group on High Blood Pressure in Children and Adolescents [22].

### 2.3. Analysis of cMVs by Flow Cytometry

The plasma samples were thawed in melting ice and vortexed before a new centrifugation at 2500 × g for 10 minutes at room temperature (RT). Plasma from the upper part of the vial was pipetted to a second vial, and the cMVs were washed and carefully separated from plasma by a two-step high-speed centrifugation at 20 000 × g for 30 minutes at 20°C. The cMV pellets were added citrate-phosphate-buffered saline (citrate PBS) and set up for triple-label flow cytometry. In a 96-well plate, the cMV suspension (5 *μ*L) was mixed with eight different combinations of AV^+^ labeled with allophycocyanin (APC) (5 *μ*L) with two specific monoclonal antibodies (mAb, 5 *μ*L each, see Supplementary [Supplementary-material supplementary-material-1]) diluted in Annexin Binding Buffer (ABB) (30 *μ*L). The different combinations included APC/CD142/CD61, APC/CD62P/CD62L, APC/CD146/CD62E, APC/CD309/CD34, APC/CD31/CD42b, APC/CD15/CD45, APC/CD11b/CD14, and APC/CD142/CD14. The mAb were conjugated to fluorescein isothiocyanate (FITC) or phycoerythrin (PE), or the isotype-matched control antibodies. The labeling was ended by ABB after 20-minute incubation in the dark at RT, and the samples were directly measured in the Auto Collect mode on an Accuri C6 flow cytometer (BD, Accuri® Cytometers, Inc., San Diego, CA). The flow cytometer was programmed to collect forward scatter (FSC), side scatter (SSC), and fluorescence data in the logarithmic scale, and all test tubes had 2 minutes of acquisition at a flow rate of 14 *μ*L/minute. cMVs were quantified and characterized according to their binding to AV^+^ and cell-specific mAb (see Supplemental [Supplementary-material supplementary-material-1]). The buffers were freshly made every day and filtered (0.2 *μ*m) to minimize the background noise. To correct for autofluorescence, signals obtained with cMVs in a calcium-free buffer (PBS) were utilized. The size threshold was accomplished with the Megamix-Plus FSC, a mix of beads with cMV-equivalent sizes: 0.1 *μ*m, 0.3 *μ*m, 0.5 *μ*m, and 0.9 *μ*m (BioCytex, Marseille, France), as previously described [23]. To correctly identify positive events, limits of fluorescence were determined by samples incubated with the isotype-matched control antibodies.

The total number of cMVs per *μ*L of platelet-free plasma was computed with Nieuwland's procedure [24], as previously explained [23]. The BD software version 1.0.264.21 (Accuri® Cytometers, Inc.) was utilized to analyze data.

### 2.4. Statistical Analysis

Clinical and demographic characteristics are presented as medians (25^th^ and 75^th^ percentiles) or means (±standard deviation (SD)) according to the distribution of data. Categorical data are reported as numbers (%). The AV^+^ cMVs were nonnormally distributed and expressed as medians (25^th^ and 75^th^ percentiles). To compare continuous and categorical variables, Mann-Whitney *U* test, independent Student *t*-test, and chi-square tests were used as appropriate. Correlation analyses were performed by Spearman's rho, and Bonferroni corrections were applied for multiple comparisons. Multivariate linear regression models were utilized to adjust for covariates. Statistical analyses were performed using the IBM© SPSS© Statistics version 25.0 (IBM Corp., New York, NY, USA). *p* values ≤ 0.05 were considered statistically significant.

## 3. Results

The clinical characteristics for the children and adolescents with type 1 diabetes and controls, at inclusion and the five-year follow-up, are described in Table 1. There were no significant differences in age and sex. The median disease duration in the diabetes group was five years at inclusion and ten years at follow-up, and at inclusion, they had significantly higher levels of C-reactive protein (CRP), HbA1c, and anthropometric measures as well as total and low-density lipoprotein (LDL) cholesterol. At the five-year follow-up, similar group differences were present, in addition to significantly lower levels of S-creatinine and higher U-ACR in the subjects with type 1 diabetes. Use of oral contraceptives (OC) was similar in both groups; however, it increased by approximately 40% after five years.

### 3.1. AV^+^ cMVs in Type 1 Diabetes and Healthy Controls

The AV^+^ cMVs measured in both study groups at inclusion and at follow-up included total AV^+^ cMVs and AV^+^ cMVs derived from platelets (CD61^+^, CD61^+^/CD142^+^, CD42b^+^, CD31^+^/CD42b^+^, and CD62P^+^), endothelial cells (CD146^+^, CD62E^+^, CD146^+^/CD62E^+^, CD309^+^, CD309^+^/CD34^+^, and CD31^+^/CD42b^−^), platelet/endothelium (CD31^+^), pluripotent stem cells (CD34^+^), and leukocytes (CD45^+^, CD15^+^, CD45^+^/CD15^+^, CD14^+^, CD14^+^/CD11b^+^, and CD14^+^/CD142^+^). The complete and unadjusted data are shown in Supplementary [Supplementary-material supplementary-material-1]. At inclusion, the levels of AV^+^/CD61^+^/CD142^+^ and AV^+^/CD15^+^ were higher and lower, respectively, in the control group compared to the type 1 diabetes group; however, the significance was lost with Bonferroni correction (*p* > 0.002 by 23 comparisons). At the five-year follow-up, total AV^+^ cMVs were significantly lower in type 1 diabetes patients compared with controls (*p* = 0.002), Bonferroni corrected. When adjusting for conventional covariates (age and sex) and for those that differed between the two study groups (body mass index (BMI), total cholesterol, and CRP), the difference in cMVs was no longer present (Table 2).

The levels of total (AV^+^) and platelet- (CD61^+^) and leukocyte- (CD45^+^) derived AV^+^ cMVs increased in both groups during the study period, in addition to AV^+^ cMVs from activated cells (CD11b^+^) in the type 1 diabetes group; however, the change was only statistically significant for total and leukocyte-derived AV^+^ cMVs in the control group with Bonferroni correction (Supplementary [Supplementary-material supplementary-material-1]).

In the type 1 diabetes group, change in (delta value of) HbA1c was not correlated with changes in AV^+^ cMVs (data not shown).

### 3.2. Correlations between AV^+^ cMVs, Glycemic Control, and Cardiovascular Risk Factors in the Type 1 Diabetes Group

Unadjusted coefficients of correlation for those AV^+^ cMVs that correlated with HbA1c or any of the cardiovascular risk factors at inclusion and five-year follow-up are shown in Supplementary [Supplementary-material supplementary-material-1].

**Figure 1:**
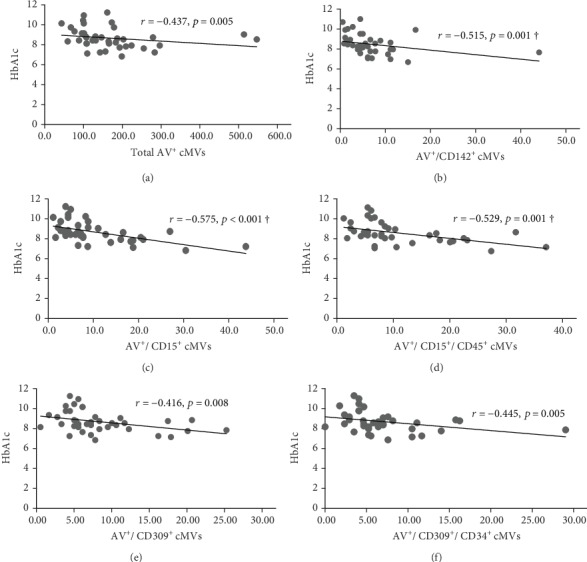
Correlations between HbA1c and AV^+^ cMVs in the type 1 diabetes group at inclusion. *r* denotes the coefficient of correlation (Spearman), and *p* indicates the significance level (*p* ≤ 0.008 with Bonferroni correction). ^†^Significant after adjustments for age, sex, total cholesterol, and CRP. Abbreviations: AV^+^: Annexin V positive; cMVs: circulating microvesicles; BMI: body mass index; CRP: C-reactive protein.

At inclusion, Bonferroni corrected (*p* = 0.008 by 6 comparisons) significant inverse correlations were observed between total (AV^+^), TF-expressing (CD142^+^), and neutrophil- (CD15^+^ and CD45^+^/CD15^+^) and endothelial-derived (CD309^+^ and CD309^+^/CD34^+^) AV^+^ cMVs and HbA1c (*r* = ‐0.437, *r* = ‐0.515, *r* = ‐0.575, *r* = ‐0.529, *r* = ‐0.416, and *r* = ‐0.445, respectively; all *p* ≤ 0.008) (Figure 1). However, when adjusted for covariates (age, sex, BMI, total cholesterol, and CRP), only the correlations between HbA1c and TF-expressing (CD142^+^) and neutrophil-derived (CD15^+^ and CD45^+^/CD15^+^) AV^+^ cMVs were still statistically significant. No significant correlations with cardiovascular risk factors were observed at inclusion.

At follow-up, no significant correlations between AV^+^ cMVs and glycemic control were observed. The inverse correlations between neutrophil-derived (CD15^+^ and CD45^+^/CD15^+^) AV^+^ cMVs and CRP (*r* = ‐0.570 and *r* = ‐0.527, respectively; both *p* ≤ 0.001) were not significant when adjusted for covariates (age, sex, BMI, total cholesterol, and HbA1c) (Table 3).

## 4. Discussion

In the present study, children and adolescents with type 1 diabetes and healthy control subjects had comparable levels of AV^+^ cMVs over a five-year period. In the type 1 diabetes group, HbA1c was associated with lower levels of TF-expressing and neutrophil derived cMVs at inclusion. However, no associations with glycemic control or cardiovascular risk factors were observed at the five-year follow-up.

The main result with similar levels of AV^+^ cMVs in children and adolescents with type 1 diabetes and controls was unexpected and contradictory to our proposed hypothesis. The lack of differences might be due to the relatively young age, short duration of diabetes, limited follow-up period, and the complete absence of comorbidities. The diabetic patients had elevated levels of several proatherogenic risk factors, but still within the normal ranges, and may not have influenced AV^+^ cMVs levels. Several previous studies have reported on elevated levels of cMVs in both type 1 and type 2 diabetes subjects compared withhealthy controls [20, 25, 26]; however, the studies have predominantly been performed in adults while the subjects in the current cohort were young and free from micro- and macrovascular complications. In a recent study on youths with type 1 diabetes compared to age- and sex-matched healthy controls, significantly higher levels of especially platelet-derived cMVs were demonstrated. However, comparison to our study may not be valid as not all patients were on regular insulin therapy and approximately one-third had vascular complications like hypertension, stroke, and impaired renal function [27]. In a cross-sectional study involving children and adolescents with type 1 diabetes, the highest levels of platelet-derived cMVs were observed in those with microvascular complications compared to without complications and healthy controls [28]. The HbA1c values in the present diabetes cohort were far above the treatment goal and further increased during the study period. High levels of HbA1c are commonly seen during puberty, and in this period of life, the body becomes more resistant to insulin and the secretion of growth hormones raises the blood glucose levels. In addition, the transition from childhood to adolescence includes challenges with disease management and poor overall glucose control [29, 30]. Nevertheless, the levels of total AV^+^ cMVs were numerically lower in the diabetes subjects, and one might speculate if it results from the effects of frequent acute insulin exposure on the vascular endothelium, beyond the glucose-lowering ability. In insulin-sensitive subjects, the vasculoprotective effects of insulin are favored with increased release of the potent vasodilator nitric oxide (NO) via the phosphatidylinositol 3-kinase (PI3K) pathway [31]. Insulin has further been shown to inhibit TF expression on monocytes and monocyte-derived microvesicles in a cyclic adenosine monophosphate- and Ca^2+^-dependent manner [32].

Recently, a Swedish study found significantly higher levels of cMVs in the type 1 diabetes group compared with healthy controls. However, PS-negative cMVs dominated in the diabetes subjects, and the authors suggested a nonproportional relationship between the transport of PS to the outer leaflet of the membrane and the accelerated release of cMVs in diabetes, which might endorse the present results [25]. In addition, inflammation, which is more reflected by AV^−^ cMVs, seems to play a more active role than thrombosis in the current cohort [33], which further supports the lack of differences in procoagulant AV^+^ cMVs.

In the type 1 diabetes group, HbA1c was associated with lower levels of TF-expressing and neutrophil-derived AV^+^ cMVs at inclusion. Although a substantial part of the published literature reports on a positive association between the levels of cMVs and fasting glucose and HbA1c [15, 18, 27], some studies do not show such a relationship [34]. The inverse associations in the present study might have a physiological explanation, by extended and frequent need of insulin therapy in those with higher HbA1c. Nevertheless, no significant associations with glycemic control were observed after ten years of disease duration. The longitudinal changes with increased levels of total and leukocyte-derived cMVs, also present in the diabetes subjects before Bonferroni correction, might therefore not be explained by hyperglycemia, rather partially caused by increased use of OC, known to promote a procoagulant state, in addition to advancing age [35].

In adults, it is generally accepted that levels of cMVs are raised in the presence of cardiovascular risk factors like hypertension, dyslipidemia, diabetes, and obesity [18, 36–38]. In the current study, no associations with cardiovascular risk factors were seen in the diabetes group. Although the levels of LDL, CRP, and BMI were significantly higher in this group compared with the healthy control subjects, the levels were still within the normal ranges. In a previous study on type 1 diabetes children and adolescents, positive correlations between platelet-derived cMVs, cardiovascular risk factors, and carotid intima-media thickness were demonstrated and the authors discussed platelet-derived cMVs to be markers of microvascular complications and subclinical atherosclerosis [28]. Thus, our findings might again be explained by the absence of vascular complications.

Analyses of cMVs are hampered with great variation, and the International Society on Thrombosis and Haemostasis Vascular Biology Standardization Subcommittee recommends to standardize the preanalytical variables to achieve less interlaboratory variance. The centrifugation is the main condition affecting the cMV count, and a double centrifugation at 2500 × g for 15 minutes in room temperature is recommended [39]. In the absence of a standardized method, we introduced a second centrifugation after thawing of the plasma samples in order to assure a complete cell/dust removal. We assured the same yield of purification between the various patient samples because all samples were handled following the same procedure. In addition, all cMV analyses from different samples from the same patient were performed the same day to avoid variability within each patient. Storage and centrifugation at 4°C might cause platelet disruption and cold activation of coagulation factor VII; however, in a recent study investigating storage of sodium citrate tubes at 4°C, no significant increase in release of platelet-derived cMVs was seen [40].

A possible limitation of the current study is that the cMV analyses have been performed on procoagulant AV^+^ cMVs only, while AV^−^ cMVs have not been taken into account, which might have underestimated the results in this relatively young population with type 1 diabetes. It should also be emphasized that our diabetes group had been on intensive insulin treatment for at least ten years.

## 5. Conclusions

In the present study, children and adolescents with type 1 diabetes show similar levels of AV^+^ cMVs as healthy control subjects and limited associations with hyperglycemia and cardiovascular risk factors. Thus, our young subjects with type 1 diabetes seem to have preserved vascular homeostasis and absence of prothrombotic cMVs after ten years of intensive insulin treatment.

## Figures and Tables

**Table 1:** Clinical data according to study groups at inclusion and at five-year follow-up (median (25^th^ and 75^th^ percentiles)).

	Inclusion			Five-year follow-up		
	Type 1 diabetes (*n* = 40)	Controls (*n* = 40)	*p*	Type 1 diabetes (*n* = 40)	Controls (*n* = 40)	*p*
Age (years)^†^	14 (9, 19)	13 (9, 20)	0.645	19 (14, 24)	18 (14, 25)	0.662
Females, *n* (%)	23 (58)	24 (60)	1.000	23 (58)	24 (60)	1.000
Oral contraceptives, *n* (%)^ǂ^						
Users	1 (4)	3 (13)	0.444	11 (48)	14 (58)	0.543
Nonusers	14 (61)	10 (42)	—	11 (48)	8 (33)	—
Not answered	8 (35)	11 (46)	—	1 (4)	2 (8)	—
Smokers, *n* (%)	0 (0)	0 (0)	—	0 (0)	1 (3)	
CRP (mg/L)	0.57 (0.30, 2.41)	0.33 (0.25, 0.67)	0.014	1.87 (0.41, 5.33)	0.56 (0.26, 2.52)	0.097
Diabetic measures						
Years of diabetes	5.0 (2.2, 9.2)	—	—	10.1 (7.2, 14.2)	—	—
HbA1c (mmol/mol)	69.4 (62.8, 75.9)	35.5 (33.3, 37.7)	<0.001	79.2 (74.9, 92.3)	34.4 (32.2, 36.6)	<0.001
(%)	8.5 (7.9, 9.1)	5.4 (5.2, 5.6)	<0.001	9.4 (8.7, 10.5)	5.4 (5.1, 5.5)	<0.001
Insulin pump, *n* (%)^$^	14 (38)	—	—	15 (50)	—	—
Anthropometric measures						
BMI (kg/m^2^)^†^	21.1 (3.6)	19.0 (2.5)	0.003	24.5 (4.2)	22.1 (2.7)	0.002
Weight (kg)^†^	54.5 (15.0)	48.9 (12.5)	0.076	71.5 (13.4)	65.8 (11.9)	0.047
Waist circumference (cm)^†^	71.2 (9.4)	66.8 (7.0)	0.022	79.5 (8.3)	74.9 (7.8)	0.015
SBP (mmHg)	100 (95, 105)	95 (90, 105)	0.073	110 (107, 120)	110 (100, 115)	0.231
DBP (mmHg)	55 (52, 65)	58 (50, 62)	0.700	72 (65, 78)	68 (60, 70)	0.002
Lipid status						
Total cholesterol (mmol/L)	4.70 (4.30, 5.20)	4.20 (3.80, 4.50)	0.002	4.60 (4.00, 5.08)	4.20 (3.60, 4.80)	0.040
LDL (mmol/L)	2.63 (1.97, 2.96)	2.29 (1.82, 2.57)	0.029	2.45 (2.04, 2.94)	2.29 (1.96, 2.67)	0.349
HDL (mmol/L)	1.73 (1.47, 1.97)	1.56 (1.38, 1.92)	0.153	1.62 (1.28, 1.97)	1.47 (1.32, 1.70)	0.202
TG (mmol/L)	0.73 (0.53, 0.99)	0.62 (0.46, 0.78)	0.059	0.90 (0.69, 1.34)	0.79 (0.70, 0.94)	0.108
Kidney function						
S-creatinine (*μ*mol/L)^†^	54 (10)	55 (9)	0.516	66 (10)	74 (11)	0.002
U-ACR (mg/mmol)	0.70 (0.60, 1.20)	0.60 (0.37, 1.23)	0.101	0.50 (0.28, 2.03)	0.3 (0.1, 0.83)	0.039

^†^Mean (range or SD), ^ǂ^% among the females within each study group, ^$^% of the T1D children that answered the question at inclusion (*n* = 37) and at five-year follow-up (*n* = 30). *p* denotes the significance level. Abbreviations: CRP: C- reactive protein; BMI: body mass index; SBP: systolic blood pressure; DBP: diastolic blood pressure; LDL: low-density lipoprotein cholesterol; HDL: high-density lipoprotein cholesterol; TG: triglycerides; U-ACR: urinary albumin to creatinine ratio.

**Table 2:** The significance of having type 1 diabetes according to levels of total AV^+^ cMVs at five-year follow-up.

Exposure	Outcome	*β*	95% CI	*p*
Type 1 diabetes^†^	lnAV^+^ cMVs	-0.351	-0.527, -0.131	0.001
Type 1 diabetes^ǂ^	lnAV^+^ cMVs	-0.315	-0.501, -0.083	*0.007*

*β* is the linear regression coefficient; *p* denotes the probability of significance (*p* ≤ 0.002 with Bonferroni correction). ^†^Unadjusted. ^ǂ^Adjusted for age, sex, BMI, ln total cholesterol, and lnCRP. Abbreviations: AV^+^: Annexin V positive; cMVs: circulating microvesicles; ln: natural logarithm; CI: confidence interval; BMI: body mass index; CRP: C-reactive protein.

**Table 3:** The association between CRP and neutrophil-derived AV^+^ cMVs in the type 1 diabetes group at follow-up.

Exposure	Outcome	*β*	95% CI	*p*
lnCRP^†^	lnAV^+^/CD15^+^	-0.566	-0.434, -0.150	<0.001
lnCRP^ǂ^	lnAV^+^/CD15^+^	-0.464	-0.417, -0.061	*0.010*
lnCRP^†^	lnAV^+^/CD15^+^/CD45^+^	-0.539	-0.406, -0.128	<0.001
lnCRP^ǂ^	lnAV^+^/CD15^+^/CD45^+^	-0.448	-0.398, -0.045	*0.015*

*β* is the linear regression coefficient; *p* denotes the probability of significance (*p* ≤ 0.008 with Bonferroni correction). ^†^Unadjusted. ^ǂ^Adjusted for age, sex, BMI, ln total cholesterol, and HbA1c. Abbreviations: CRP: C-reactive protein; AV^+^: Annexin V positive; cMVs: circulating microvesicles; ln: natural logarithm; CI: confidence interval; BMI: body mass index.

## Data Availability

The clinical and cMVs data used to support the findings of this study are available from the corresponding author upon request.

## Supplementary Materials

Supplementary Materials Supplemental Figure 1: shows plots (a-e) from the identification, characterization and quantification of AV^+^ cMVs with the Accuri C6 flow cytometer. Supplementary Table 1: presents the cell surface molecules and equivalent mAb used for identification and characterization of AV^+^ cMVs. Supplementary Table 2: shows the number of AV^+^ cMVs/*μ*L platelet-free plasma, according to study groups at inclusion and five-year follow-up. Differences between groups at both time points, changes within groups, and differences in changes between groups have been calculated. Supplementary Table 3: presents AV^+^ cMVs that correlated (Spearman's rho) with glycemic control or cardiovascular risk factors in the type 1 diabetes group at inclusion and five-year follow-up.
