# Supplementary material for: Annexin V+ Microvesicles in Children and Adolescents with Type 1 Diabetes: A Prospective Cohort Study
Source: J Diabetes Res. 2020 Mar 30;2020:7216863. doi: 10.1155/2020/7216863 (PMC7149325; doi:10.1155/2020/7216863)
Supplement: Supplementary Materials — Supplemental Figure 1: shows plots (a-e) from the identification, characterization and quantification of AV+ cMVs with the Accuri C6 flow cytometer. Supplementary Table 1: presents the cell surface molecules and equivalent mAb used for identification and characterization of AV+ cMVs. Supplementary Table 2: shows the number of AV+ cMVs/μL platelet-free plasma, according to study groups at inclusion and five-year follow-up. Differences between groups at both time points, changes within groups, and differences in changes between groups have been calculated. Supplementary Table 3: presents AV+ cMVs that correlated (Spearman's rho) with glycemic control or cardiovascular risk factors in the type 1 diabetes group at inclusion and five-year follow-up. [file 7216863.f1.pdf]

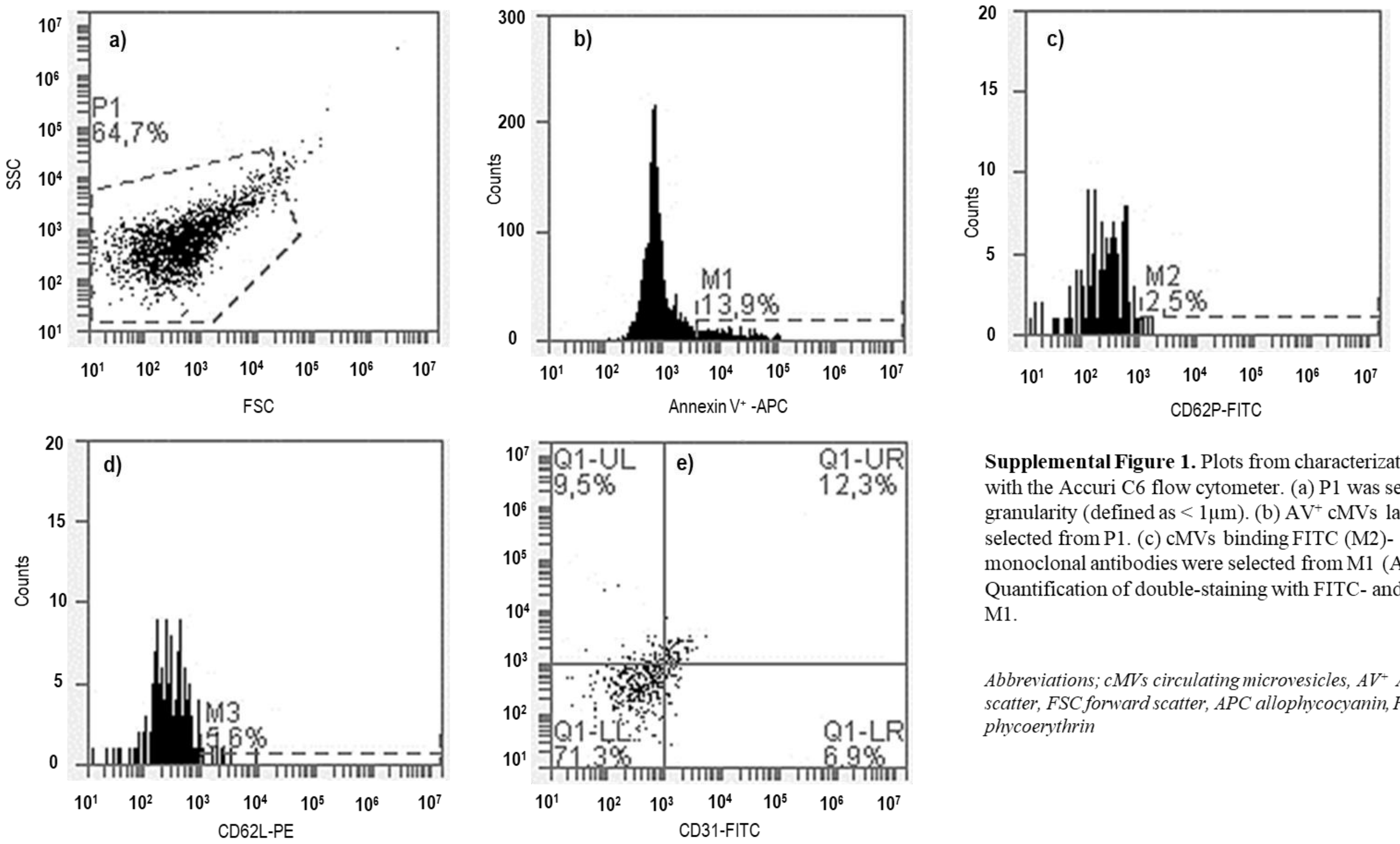

Supplemental Figure 1. Plots from characterization and quantification of cMVs with the Accuri C6 flow cytometer. (a) P1 was set according to cMVs size and granularity (defined as < 1µm). (b) AV^+ cMVs labeled with APC (M1) were selected from P1. (c) cMVs binding FITC (M2)- or (d) PE (M3)- labeled monoclonal antibodies were selected from M1 (AV^+ cMVs) and quantified. (e) Quantification of double-staining with FITC- and PE-labeled antibodies from M1.

Abbreviations; cMVs circulating microvesicles, AV^+ Annexin V positive, SSC side scatter, FSC forward scatter, APC allophycocyanin, FITC fluorescein isothiocyanate, PE phycoerythrin

Supplementary Table 1. Cell surface molecules for circulating microvesicle identification and characterization

mAb	Alternative name	Expression
Annexin V	PS-binding protein	Widely expressed
CD142	Tissue Factor	Widely expressed
CD61	β_3-integrin	Platelets
CD31	Platelet endothelial cell adhesion molecule	Platelets, Endothelial cells
CD62P	P-Selectin	Activated Platelets
CD42b	receptor for von Willebrand factor	Activated Platelets
CD146	Melanoma Cell Adhesion Molecule	Endothelial Cells
CD62E	E-Selectin	Endothelial Cells
CD34	Mucosialin	Progenitor and Stem Cells
CD309	vascular endothelial growth factor receptor-2	Endothelial Cells
CD45	Leukocyte Common Antigen	Leukocytes
CD11b	Lymphocyte function-associated antigen 1	Leukocytes
CD62L	L-Selectin	Leukocytes
CD14	LPS-receptor	Macrophages, monocytes
CD15	Sialyl-Lewis X	Neutrophils, eosinophils and monocytes

Abbreviations; mAb monoclonal antibody, PS phosphatidylserine, LPS lipopolysaccharide, CD cluster of differentiation

Supplementary Table 2. AV^+ cMVs according to groups at inclusion and five-year follow-up (median (25th, 75th percentiles)

	**Inclusion**			**5 years follow-up**			
cMVs/µl PFP	T1D (n= 40)	Controls (n=40)	p	T1D (n= 40)	Controls (n=40)	p	Δp
Total AV^+	142.89 (96.65, 190.40)	155.60 (112.80, 232.99)	0.252	158.74^{*} (137.17, 213.91)	248.06^{***} (171.88, 349.97)	0.002	0.133
Platelet derived							
$CD61^+$	115.45 (74.94, 158.05)	123.06 (82.55, 193.29)	0.317	129.98^{*} (102.18, 170.91)	188.61^{**} (129.15, 240.74)	*0.008*	0.544
$CD61^+/CD142^+$	1.66 (0.55, 2.77)	2.77 (1.11, 6.08)	0.017	1.11 (0.55, 3.18)	2.77 (1.66, 6.64)	*0.004*	0.935
$CD42b^+$	33.46 (18.39, 56.83)	29.87 (19.50, 67.75)	0.577	37.89 (22.54, 65.68)	47.84 (30.39, 58.90)	0.197	0.985
$CD31^+/CD42b^+$	17.43 (10.51, 32.77)	21.85 (13.27, 41.21)	0.225	21.67 (13.27, 35.81)	27.10 (16.39, 33.19)	0.441	0.441
$CD62P^+$	2.21 (1.11, 3.32)	2.21 (1.11, 5.25)	0.228	2.21 (1.66, 3.32)	3.04 (1.66, 4.29)	0.142	0.736
Endothelial derived							
$CD146^+$	0.55 (0.00, 1.11)	0.55 (0.00, 1.54)	0.684	0.55 (0.00, 1.11)	0.55 (0.00, 1.11)	0.618	0.911
$CD62E^+$	7.74 (5.04, 11.61)	9.68 (5.12, 16.92)	0.384	8.85 (4.98, 13.00)	10.23 (7.88, 18.81)	0.120	0.453
$CD146^+/CD62E^+$	0.00 (0.00, 1.11)	0.00 (0.00, 0.79)	0.916	0.00 (0.00, 0.79)	0.55 (0.00, 0.97)	0.086	0.444
$CD309^+$	6.91 (4.98, 10.92)	7.62 (4.42, 15.63)	0.510	6.64 (3.46, 9.40)	8.85 (4.70, 16.45)	*0.044*	0.194
$CD309^+/CD34^+$	5.11 (3.87, 7.19)	6.91 (2.77, 10.37)	0.550	5.53 (2.90, 7.19)	6.64 (2.8, 9.26)	0.278	0.743
$CD31^+/CD42b^-$	8.57 (4.98, 19.22)	13.27 (8.41, 20.19)	0.097	14.66 (10.04, 21.71)	16.87 (9.54, 23.09)	0.319	0.541
Platelet and endothelium derived							
$CD31^+$	30.62 (16.32, 47.70)	37.33 (21.16, 59.73)	0.201	36.50 (28.21, 50.06)	42.59 (29.18, 55.72)	0.242	0.433
Leukocyte derived							
$CD45^+$	53.65 (39.96, 71.61)	61.39 (51.44, 84.62)	0.070	64.99^{**} (51.16, 84.35)	80.75^{***} (57.66, 96.79)	*0.050*	0.900
$CD15^+$	7.47 (4.42, 16.45)	5.25 (2.77, 8.85)	0.019	7.19 (5.53, 14.24)	7.74 (4.01, 11.06)	0.630	0.064
$CD45^+/CD15^+$	7.19 (4.98, 14.66)	5.53 (3.32, 9.54)	0.067	7.19 (5.53, 14.10)	6.64 (4.46, 11.06)	0.583	0.233

CD14$^+$	2.21 (1.66, 3.73)	2.77 (1.66, 5.53)	0.178	2.21 (1.66, 3.87)	3.32 (2.21, 4.98)	0.112	0.718
CD14$^+$/CD11b$^+$	1.66 (0.55, 2.73)	1.66 (0.55, 4.42)	0.179	1.66 (0.55, 2.63)	2.21 (0.90, 4.42)	0.101	0.762
CD14$^+$/CD142$^+$	1.11 (0.55, 2.21)	1.66 (0.10, 3.87)	0.406	1.11 (0.55, 2.77)	1.66 (0.55, 3.18)	0.247	0.969
Pluripotent stemcell derived							
CD34$^+$	30.42 (22.82, 44.39)	34.29 (23.06, 47.98)	0.433	32.91 (24.61, 48.12)	38.44 (32.63, 48.95)	0.218	0.885
Activated cell derived							
CD62L	4.70 (3.36, 8.30)	4.98 (3.87, 11.06)	0.446	4.98 (3.32, 7.05)	5.53 (3.87, 9.81)	0.143	0.912
CD142$^+$	4.98 (3.04, 8.57)	8.30 (3.87, 18.11)	0.020	5.53 (3.32, 10.37)	9.96 (5.53, 17.28)	*0.013*	0.825
CD11b$^+$	14.93 (7.99, 24.47)	19.63 (12.56, 34.98)	0.048	17.70* (12.31, 28.21)	24.61 (17.32, 33.19)	*0.018*	0.992

p denotes the probability for group differences ($p \leq 0.002$ with Bonferroni correction)

Δp denotes the probability for differences in changes between the groups calculated on delta values

Changes within groups are indicated by *(* < 0.05, **< 0.01 and *** ≤ 0.001)

Abbreviations: AV$^+$ Annexin V positive, cMVs circulating microvesicles, PFP platelet free plasma, T1D type 1 diabetes

Supplementary Table 3. AV^{+} cMVs that associated with glycemic control or traditional cardiovascular risk factors in the type 1 diabetes group at inclusion and five-year follow-up

		**Inclusion**						**Five-year follow-up**					
cMVs		HbA1c	Age	BMI	SBP	LDL	CRP	HbA1c	Age	BMI	SBP	LDL	CRP
AV^{+}	r	-0.437	-0.035	-0.281	0.060	0.133	-0.039	0.134	-0.405	-0.357	-0.311	-0.056	-0.159
	p	0.005	0.832	0.080	0.719	0.420	0.810	0.410	*0.010*	*0.024*	*0.050*	0.732	0.334
AV^{+}/ CD61^{+}	r	-0.311	-0.034	-0.321	0.046	0.103	-0.032	0.215	-0.336	-0.342	-0.228	-0.090	-0.122
	p	0.054	0.835	*0.043*	0.781	0.532	0.844	0.183	*0.034*	*0.031*	0.156	0.580	0.459
AV^{+}/ CD45^{+}	r	-0.333	-0.194	-0.179	0.055	0.057	0.041	0.132	-0.184	-0.192	-0.030	0.201	-0.369
	p	*0.039*	0.231	0.269	0.740	0.731	0.804	0.416	0.255	0.236	0.854	0.214	*0.021*
AV^{+}/ CD15^{+}	r	-0.575	-0.060	-0.121	0.006	0.218	-0.018	-0.003	-0.143	-0.216	0.092	0.100	-0.570
	p	< 0.001	0.712	0.459	0.971	0.182	0.910	0.987	0.379	0.180	0.571	0.540	< 0.001
AV^{+}/ CD45^{+}/CD15^{+}	r	-0.529	-0.118	-0.140	-0.040	0.182	-0.022	-0.018	0.007	-0.126	0.134	0.145	-0.527
	p	0.001	0.468	0.387	0.808	0.266	0.894	0.912	0.967	0.439	0.408	0.372	0.001
AV^{+}/ CD11b^{+}	r	-0.295	0.024	-0.236	0.140	-0.018	-0.076	-0.072	-0.178	-0.168	0.035	0.006	-0.349
	p	0.068	0.883	0.143	0.396	0.913	0.640	0.659	0.272	0.299	0.832	0.971	*0.030*
AV^{+}/ CD14^{+}	r	-0.305	0.236	-0.003	0.186	0.111	-0.211	-0.333	-0.014	-0.087	-0.119	-0.394	-0.147
	p	0.059	0.143	0.986	0.257	0.500	0.192	0.036	0.933	0.591	0.466	*0.012*	0.371
AV^{+}/ CD142^{+}	r	-0.515	0.170	-0.082	0.092	0.207	-0.130	-0.136	0.062	-0.234	0.139	-0.217	-0.277
	p	0.001	0.296	0.616	0.578	0.207	0.424	0.404	0.705	0.147	0.394	0.178	0.088
AV^{+}/ CD14^{+}/CD142^{+}	r	-0.319	0.212	0.129	0.238	0.193	-0.015	-0.146	0.154	-0.037	0.270	-0.333	-0.168
	p	*0.048*	0.189	0.426	0.145	0.240	0.925	0.370	0.342	0.819	0.092	0.036	0.306

AV^+/ $CD309^+$	r	-0.416	0.118	-0.095	0.171	-0.081	0.096	0.000	-0.134	-0.310	-0.078	-0.001	-0.195
	p	0.008	0.468	0.561	0.298	0.622	0.554	0.998	0.411	0.051	0.634	0.997	0.235
AV^+/ $CD309^+$/$CD34^+$	r	-0.445	0.064	-0.167	0.139	-0.113	0.030	-0.085	-0.060	-0.297	-0.043	-0.054	-0.211
	p	0.005	0.693	0.304	0.399	0.495	0.853	0.601	0.714	0.063	0.792	0.739	0.198
AV^+/ $CD31^+$	r	-0.208	-0.056	-0.178	0.097	0.150	0.052	0.126	-0.307	-0.349	-0.097	-0.189	-0.269
	p	0.204	0.731	0.273	0.556	0.363	0.748	0.439	0.054	0.028	0.550	0.244	0.098
AV^+/ $CD31^+$/ $noCD42b^+$	r	-0.249	-0.063	-0.211	-0.063	0.110	0.116	-0.002	-0.210	-0.368	-0.294	0.062	-0.382
	p	0.126	0.701	0.192	0.703	0.505	0.476	0.991	0.194	*0.020*	0.065	0.706	*0.016*

r denotes the coefficient of correlation (Spearman's rho), p denotes the probability of the correlation ($p \leq 0.008$ with Bonferroni correction).

Abbreviations; AV^+ cMVs Annexin V positive circulating microvesicles, BMI body mass index, SBP systolic blood pressure, LDL low density lipoprotein cholesterol, CRP C-reactive protein

STROBE Statement—checklist of items that should be included in reports of observational studies

	Item No.	Recommendation	Page No.	Relevant text from manuscript
**Title and abstract**	1	(*a*) Indicate the study's design with a commonly used term in the title or the abstract	1, 2	
		(*b*) Provide in the abstract an informative and balanced summary of what was done and what was found	2	
**Introduction**				
Background/rationale	2	Explain the scientific background and rationale for the investigation being reported	3, 4	
Objectives	3	State specific objectives, including any prespecified hypotheses	4	
**Methods**				
Study design	4	Present key elements of study design early in the paper	4	
Setting	5	Describe the setting, locations, and relevant dates, including periods of recruitment, exposure, follow-up, and data collection	4, 5	
Participants	6	(*a*) *Cohort study*—Give the eligibility criteria, and the sources and methods of selection of participants. Describe methods of follow-up<br>*Case-control study*—Give the eligibility criteria, and the sources and methods of case ascertainment and control selection. Give the rationale for the choice of cases and controls<br>*Cross-sectional study*—Give the eligibility criteria, and the sources and methods of selection of participants	4	
		(*b*) *Cohort study*—For matched studies, give matching criteria and number of exposed and unexposed<br>*Case-control study*—For matched studies, give matching criteria and the number of controls per case	-	

Variables	7	Clearly define all outcomes, exposures, predictors, potential confounders, and effect modifiers. Give diagnostic criteria, if applicable	7, 8, 9
Data sources/ measurement	8*	For each variable of interest, give sources of data and details of methods of assessment (measurement). Describe comparability of assessment methods if there is more than one group	4-6
Bias	9	Describe any efforts to address potential sources of bias	-
Study size	10	Explain how the study size was arrived at	4, 5

Continued on next page

Quantitative variables	11	Explain how quantitative variables were handled in the analyses. If applicable, describe which groupings were chosen and why	6, 7
Statistical methods	12	(*a*) Describe all statistical methods, including those used to control for confounding	7
		(*b*) Describe any methods used to examine subgroups and interactions	-
		(*c*) Explain how missing data were addressed	-
		(*d*) *Cohort study*—If applicable, explain how loss to follow-up was addressed<br>*Case-control study*—If applicable, explain how matching of cases and controls was addressed<br>*Cross-sectional study*—If applicable, describe analytical methods taking account of sampling strategy	-
		(*e*) Describe any sensitivity analyses	-
**Results**			
Participants	13*	(a) Report numbers of individuals at each stage of study—eg numbers potentially eligible, examined for eligibility, confirmed eligible, included in the study, completing follow-up, and analysed	4
		(b) Give reasons for non-participation at each stage	-
		(c) Consider use of a flow diagram	-
Descriptive data	14*	(a) Give characteristics of study participants (eg demographic, clinical, social) and information on exposures and potential confounders	18, 19<br>7
		(b) Indicate number of participants with missing data for each variable of interest	-
		(c) *Cohort study*—Summarise follow-up time (eg, average and total amount)	
Outcome data	15*	*Cohort study*—Report numbers of outcome events or summary measures over time	Supplementary Table 2.

		Case-control study—Report numbers in each exposure category, or summary measures of exposure	
		Cross-sectional study—Report numbers of outcome events or summary measures	
Main results	16	(*a*) Give unadjusted estimates and, if applicable, confounder-adjusted estimates and their precision (eg, 95% confidence interval). Make clear which confounders were adjusted for and why they were included	Supplementary Table 2 and 3, page 20, 21 22, 8
		(*b*) Report category boundaries when continuous variables were categorized	-
		(*c*) If relevant, consider translating estimates of relative risk into absolute risk for a meaningful time period	-
